# Understanding Pain in Osteochondral Lesions of the Talus: A Cross-Sectional, CT‑Based Analysis Showing Limited and Inconsistent Associations With Pain

**DOI:** 10.1177/10711007251413568

**Published:** 2026-03-03

**Authors:** Julian J. Hollander, Inger N. Sierevelt, Jason A. H. Steman, Juliëtte H. M. Pijnacker, Kaj S. Emanuel, The Amsterdam Ankle Cartilage Team, Nathanael O. Agyeman-Prempeh, Gino M. M. J. Kerkhoffs, Sjoerd A. S. Stufkens

**Affiliations:** 1 Department of Orthopaedic Surgery and Sports Medicine, Amsterdam UMC, Location AMC, University of Amsterdam, the Netherlands; 2 Amsterdam Movement Sciences, Programs Sports and Musculoskeletal Health, the Netherlands; 3 Academic Center for Evidence based Sports medicine (ACES), Amsterdam UMC, the Netherlands; 4 Amsterdam Collaboration for Health and Safety in Sports (ACHSS), International Olympic Committee (IOC) Research Center, Amsterdam UMC, the Netherlands; 5 Department of Orthopedic Surgery, Xpert Clincs, Amsterdam, the Netherlands; 6 Department of Orthopedic Surgery, Spaarne Gasthuis Academie, Haarlem, the Netherlands

**Keywords:** osteochondral lesion, talus, OLT, lesion characteristics, ankle, pain

## Abstract

**Background:**

Patients with an osteochondral lesion of the talus (OLT) often present with deep ankle pain, but the direct relationship between structural damage and the perceived pain remains unclear. The aim of this study was therefore to determine whether pain is associated with demographic and radiologic (computed tomography [CT]-only) lesion characteristics.

**Methods:**

This cross-sectional study was conducted in patients with symptomatic OLTs at a tertiary referral academic hospital. The primary outcome was the Pain subscale of the Foot and Ankle Outcome Score (FAOS). Secondary outcomes included the Numeric Rating Scale (NRS) for pain at rest and during walking, as well as the other FAOS subscales. Associations with patient demographics, lesion size, morphology, and location were assessed using univariate linear regression, followed by multivariate linear regression with backward selection.

**Results:**

A total of 310 patients were included. In the final multivariate model for the FAOS Pain subscale, higher age (β = −0.18, *P* = .04) and smoking (β = −8.70, *P* < .001) were significantly associated with worse pain scores. Lesion morphology characterized by the presence of an osteochondral fragment was associated with lower NRS pain scores during walking. For NRS pain at rest, worse scores were significantly associated with female sex, higher body mass index, smoking, non-primary lesion nature, and greater lesion depth.

**Conclusion:**

No association was found between CT-based radiologic lesion characteristics and patient-reported pain as measured by the FAOS Pain subscale. In secondary analyses, some lesion characteristics showed associations, but these were limited and directionally inconsistent. These findings suggest structural damage alone does not seem to fully explain the patient’s pain. Given the CT‑only approach and other design constraints, further research is warranted.

## Graphical abstract

**Graphical Abstract:**
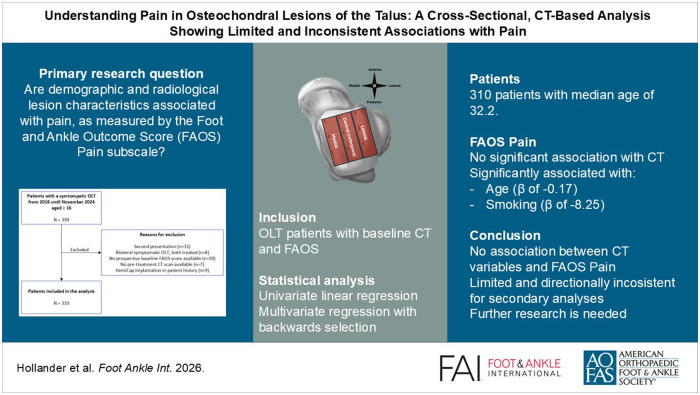
This is a visual representation of the abstract.

**Level of Evidence:** Level III, therapeutic.

## Introduction

Patients with an osteochondral lesion of the talus (OLT) often have a history of trauma, such as ankle sprain, fracture, syndesmotic injury, or chronic lateral ankle instability.^1-5^ Their symptoms are typically described as deep ankle pain during physical activity, which can substantially impair quality of life by limiting daily function and participation in sports.^6^

The pathophysiological mechanisms underlying this deep ankle pain remain poorly understood. Several hypotheses have been proposed, most notably the involvement of nerve endings within the subchondral bone.^7^ Another theory suggests that increased intra-articular synovial fluid pressure infiltrates and stimulates these nerve fibers, thereby contributing to pain.^8^ From this perspective, radiologic lesion characteristics—such as size, morphology, and location—may be expected to influence pain severity. For example, lesion location could alter synovial fluid dynamics, whereas larger lesions may involve more subchondral bone and generate increased nociceptive input.

Nevertheless, pain perception is inherently multifactorial, influenced not only by biological but also by psychological and social factor.^9,10^ A comprehensive biopsychosocial framework is therefore needed to understand the pain experience in OLT patients. If it would be possible to predict pain in OLT patients, this would allow for more targeted treatment decisions and potentially reduce the need for invasive procedures.

Accordingly, the primary aim of this study was to investigate whether demographic and radiologic lesion characteristics are associated with pain, as measured by the Foot and Ankle Outcome Score (FAOS) Pain subscale. Secondary analyses explored associations between lesion characteristics and the numeric rating scale (NRS) for pain at rest and during walking, as well as the other FAOS subscales. It was hypothesized that there is a small association between pain and radiologic computed tomography (CT) characteristics.

## Materials and Methods

### Study Design

The present study is a retrospective analysis of prospectively collected data and was conducted at an academic tertiary referral center in Amsterdam, The Netherlands. The study was approved by the Medical Ethics Committee (reference number: W14_237#14.17.0288) and was conducted in accordance with the Declaration of Helsinki.

### Patient Selection

All patients who presented to the Orthopaedic Department of the Amsterdam UMC between January 2017 and October 2024 for treatment of a symptomatic osteochondral lesion of the talus were prospectively included. Patients with concomitant conditions that could affect pain reporting—such as rheumatoid arthritis, advanced osteoporosis, malignancy, or infection—were excluded. Further exclusion criteria were incomplete baseline data (eg, missing clinical characteristics or absence of a baseline computed tomography [CT] scan).

Patients who underwent multiple treatments were included for the first presentation in order to ensure independence of observations. To avoid potential confounding from contralateral disease, patients with bilateral symptomatic lesions who received treatment for both ankles were excluded.

### Data Extraction

All demographic characteristics were extracted using the electronic data capture system CASTOR by a single researcher. Radiologic variables were independently assessed by 2 researchers. For continuous variables, such as lesion size, the average of the 2 raters was used in the analysis. In the case of disagreement regarding categorical radiologic variables (eg, lesion location and morphology), consensus was sought between 2 reviewers JH and JS. If consensus could not be reached, the senior author GK or SS served as the final arbiter.

### Patient Demographics

Patient demographics included patient sex, age at baseline (ie, prior to treatment), body mass index (BMI), smoking status, and lesion nature (primary or non-primary; ie [no] surgical treatment in patient history).

Smoking status was classified as a binary variable (smoker vs non-smoker), based on the methodology used in other studies assessing this variable specifically.^11-13^

### Radiologic Characteristics

All radiologic variables were extracted from computed tomography (CT) scans prior to the start of treatment.

Osteochondral lesion size was measured using the greatest diameter (in mm) anteroposterior (AP), mediolateral (ML) and the depth. Using these measurements, the lesion surface area was calculated using an ellipsoid formula (0.79 × ML × AP; mm^2^) and lesion volume was also calculated (AP × ML × depth; mm^3^).^14^ Interobserver reliability was assessed using intraclass correlation coefficient (ICC) analysis (Supplementary Materials).

**Figure 1.**
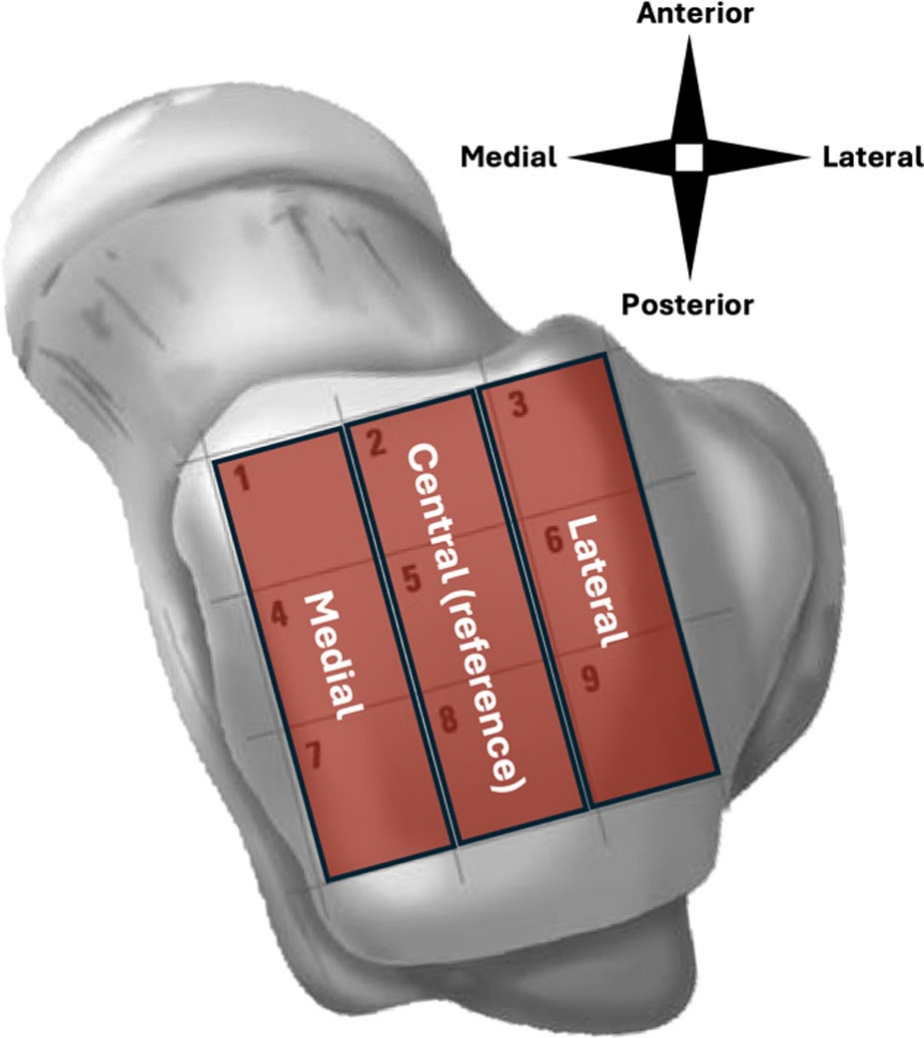
Anatomic zones.

Lesion location was determined using a 9-grid scheme and subsequently classified in being medial, central, or lateral (Figure 1).^15,16^ This categorization was done in order to increase power and interpretability. Lesion morphology was categorically defined as fragmented, crater-like, or cystic.^17^ In case of mixed location and morphology, the extracting researcher was instructed to choose the category that included the biggest part of the lesion.

### Outcome Measures

All patients received (electronic) questionnaires containing the Foot and Ankle Outcome Score with its 5 subscales (higher score = better) and the NRS of pain (lower score = better) during walking and during rest directly after their first visit at the outpatient clinic, before starting treatment.^18-20^

### Statistical Analysis

All data analyses were performed using custom-made Python scripts (version 3.11.8) with the packages Scikit-learn, Scipy, and Statsmodels.^21-24^

Dichotomous and categorical variables are presented as absolute numbers with corresponding percentages. Normally distributed continuous variables are presented as means with SDs; otherwise the median and IQR are reported. Normality was assessed by means of visual inspection of box plots and with the Shapiro-Wilk test.

To identify factors that were associated with the primary outcome (ie, the FAOS pain subscale), univariate linear regression for all lesion variables (lesion nature, lesion size dimensions [ie, AP, ML, and depth], lesion morphology, lesion location) was performed. Lesion surface area and volume were not used due to covariance. Additionally, patient characteristics that have been reported to influence treatment outcomes in the literature (sex,^25^ age at treatment,^26^ BMI,^27^ and smoking status^11^) were included. Variables that were significantly associated (adjusted significance level of 0.2) in the univariate linear regression were included in a multivariate linear regression analysis. Backwards selection was applied. For the primary outcome (FAOS pain subscale), model diagnostics using residual plots, Q-Q plots, and variance inflation factor (VIF) are provided in the Supplementary Materials for each imputed model (n = 20). A *P* <.05 was determined to be significant in the final model.

The same methodology was used for all secondary outcomes (ie, NRS of pain during walking, NRS of pain during rest and other FAOS subscales).

Multiple imputation using all model variables and 2 auxiliary variables (length and weight) was performed for missing data (BMI for 10 patients [3%] and smoking for 7 patients [2%]), assuming that the data were missing at random. Twenty data sets were created, and results were pooled using the Rubin rules.^28^ Additionally, a complete-case sensitivity analysis was performed (Supplementary Materials).

## Results

### Patient Selection and Characteristics

**Figure 2.**
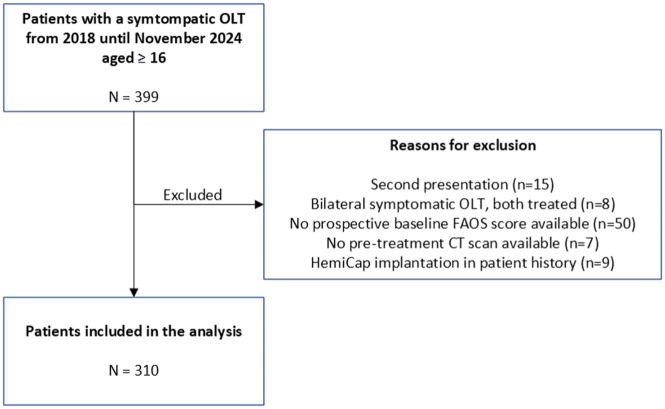
Patient selection flowchart with reason of exclusion.

A total of 310 patients were included in the present study (Figure 2). The mean age of the patients was 32.2 years (SD 12.2) and 174 patients (56%) were male. All demographic and radiologic characteristics are outlined in Table 1.

**Table 1.** Patient- and lesion characteristics.

Patient characteristics	
Age, y, mean ± SD	32.2 ± 12.2
Sex, male/female, n (%)	174 (56) / 136 (44)
BMI^a^, mean ± SD	25.8 ± 4.4
Laterality, right/left, n (%)	169 (55) / 141 (45)
Smoking, yes/no^b^, n (%)	82 (27) / 221 (73)
Lesion characteristics	
Nature, primary/non-primary, n (%)	179 (58) / 131 (42)
Lesion size	
AP, mm, mean ± SD	14.9 ± 5.2
ML, mm, mean ± SD	10.0 ± 3.6
Depth, mm, mean ± SD	7.8 ± 4.2
Surface area, mm^2^, median (IQR)	113 (74-166)
Volume, mm^3^, median (IQR)	896 (501-1830)
Lesion location, n (%)	
Medial	225 (73)
Central	14 (5)
Lateral	71 (23)
Lesion morphology, n (%)	
Crater	106 (34)
Cystic	156 (50)
Fragment	48 (15)

Abbreviations: AP, anteroposterior; BMI, body mass index; ML, mediolateral.

a Missing for 10 patients.

b Missing for 7 patients.

#### Outcomes

##### Primary outcome

The FAOS pain subscale was at baseline 56.8 (SD: 17.9). Univariate analysis showed a *P* value < .2 for age at surgery, BMI, smoking, and lesion nature. Multivariate analysis and the final model identified age at years (*P* = .048) and smoking (*P* = .0018) as predictors (Table 2). Both were negatively associated with the FAOS Pain (ie, higher age or positive smoking status associated with a worse score).

**Table 2.** Linear Regression Analysis for FAOS Pain Subscale (Higher Score Means Less Pain).

	Univariate		Multivariate		Final Multivariate Model	
Variable	β (95% CI)	*P* Value^a^	β (95% CI)	*P* Value^a^	β (95% CI)	*P* Value^a^
Sex, male	1.92 (−2.09 to 5.94)	.35				
Age at treatment, y	−0.15 (−0.31 to 0.01)	**.07**	−0.13 (−0.32 to 0.05)	.15	−0.17 (−0.34 to 0.00)	**.048**
BMI	−0.38 (−0.84 –0.07)	**.10**	−0.22 (−0.73 to 0.29)	.37		
Smoking, yes	−7.91 (−12.38 to −3.44)	**<.001**	−8.43 (−13.21 to −3.66)	**.0016**	−8.25 (−13.01 to −3.49)	**.0018**
Lesion nature, non-primary	−3.09 (−7.12 to 0.93)	**.13**	−3.53 (−7.76 to 0.70)	.10		
Lesion size						
AP, mm	0.04 (−0.35 to 0.43)	.84				
ML, mm	−0.14 (−0.70 to 0.42)	.62				
depth, mm	−0.21 (−0.68 to 0.26)	.38				
Lesion morphology (reference: crater)						
Cystic	−0.37 (−4.36 to 3.62)	.86				
Fragmentary	3.03 (−2.46 to 8.54)	.28				
Lesion location (reference: central)						
Lateral	1.39 (−3.35 to 6.13)	.57				
Medial	−0.10 (−4.57 to 4.37)	.97				

Abbreviations: AP, anteroposterior; BMI, body mass index; FAOS, foot and ankle outcome score; ML, mediolateral.

a Boldface indicates statistical significance.

The complete-case sensitivity analysis confirmed a robust analysis as comparable results in univariate analysis and the same final model were seen (Supplementary Materials) with an *R*^2^ of 0.055 and adjusted *R*^2^ of 0.048. Model diagnostic figures for all imputed data sets are available in the Supplementary Materials. No relevant violations of regression assumptions were observed, and the results were consistent across imputations. Thereby, the robustness of the final multivariate model is supported.

##### Secondary outcomes

The analysis for the secondary outcome NRS during walking found a significant association for the variable fragmentary morphology (β = −1.14, *P* = .01). For the NRS, during rest the variables sex (β = −0.69, *P* = .02), BMI (β = 0.07, *P* = .03), smoking (β = 0.69, *P* = .03), and lesion nature (β = 0.84, *P* = .005) as well as lesion size depth (β = 0.08, *P* = .02) were of importance (Table 3). These models had an *R*² of respectively 0.024 (adjusted 0.018) and 0.104 (adjusted 0.089) on the complete-case sensitivity analysis.

**Table 3.** Linear Regression Analysis for NRS During Walking and NRS During Rest (Higher Score Means More Pain).

Variable	NRS During Walking						NRS During Rest					
	Univariate		Multivariate		Final Multivariate Model		Univariate		Multivariate		Final Multivariate Model	
	β (95% CI)	*P* Value^a^	β (95% CI)	*P* Value^a^	β (95% CI)	*P* Value^a^	β (95% CI)	*P* Value^a^	β (95% CI)	*P* Value^a^	β (95% CI)	*P* Value^a^
Sex, male	−0.38 (−0.931 to 0.169)	.17	−0.46 (−1.06 to 0.14)	.12			−0.57 (−1.1 to −0.042)	**.03**	−0.70 (−1.26 to −0.13)	**.02**	−0.69 (−1.25 to −0.13)	**.02**
Age at treatment, y	0.02 (0.004 to 0.049)	**.02**	0.01 (−0.02 to 0.04)	.38			0.012 (−0.010 to 0.033)	.28				
BMI	0.07 (−0.006 to 0.130)	**.03**	0.04 (−0.04 to 0.11)	.31			0.08 (0.019 to 0.138)	**.01**	0.06 (−0.01 to 0.12)	.08	0.07 (0.01 to 0.13)	**.03**
Smoking, yes	0.26 (−0.355 to 0.884)	.40					0.58 (−0.015 to 1.177)	.06	0.73 (0.11 to 1.35)	**.02**	0.69 (0.07 to 1.31)	**.03**
Lesion nature, non-primary	0.68 (0.133 to 1.230)	**.01**	0.46 (−0.15 to 1.06)	.13			0.80 (0.272 to 1.324)	**.003**	0.69 (0.12 to 1.26)	**.02**	0.84 (0.29 to 1.39)	**.005**
Lesion size												
AP (mm)	0.01 (−0.045 to 0.061)	.78					0.02 (−0.030 to 0.072)	.43				
ML (mm)	0.05 (−0.031 to 0.123)	.24					0.03 (−0.041 to 0.107)	.38				
Depth (mm)	0.06 (−0.009 to 0.120)	**.09**	0.07 (−0.01 to 0.14)	.08			0.06 (−0.003 to 0.121)	.06	0.10 (0.02 to 0.17)	**.01**	0.08 (0.01 to 0.14)	**.02**
Lesion morphology (reference: crater)												
Cystic	0.14 (−0.402 to 0.692)	.60	−0.31 (−1.03 to 0.41)	.38	−0.21 (−0.85 to 0.43)	.50	−0.15 (−0.682 to 0.373)	.57	−0.56 (−1.23 to 0.11)	.10		
Fragmentary	−1.01 (−1.761 to −0.265)	**.008**	−0.76 (−1.69 to 0.17)	.10	−1.14 (−2.02 to −0.25)	**.01**	−0.61 (−1.336 to 0.115)	.10	−0.65 (−1.52 to 0.23)	.14		
Lesion location (reference: central)												
Lateral	−0.97 (−1.607 to −0.323)	**.003**	−0.82 (−2.31 to 0.67)	.26			−0.68 (−1.302 to −0.056)	**.03**	−0.38 (−1.80 to 1.03)	.58		
Medial	0.75 (0.137 to 1.352)	**.016**	−0.29 (−1.68 to 1.10)	.66			0.52 (−0.073 to 1.104)	.09	−0.13 (−1.45 to 1.18)	.84		

Abbreviations: AP, anteroposterior; BMI, body mass index; ML, mediolateral; NRS, numeric rating scale.

a Boldface indicates statistical significance.

The analysis of the other FAOS subscales is available in the Supplementary Materials. In the final models of these subscales, different predictors were identified per subscale: smoking and lesion nature for symptoms, age and smoking for ADL, age and lesion morphology for sports, and mediolateral lesion size and lesion morphology for the QoL subscale.

## Discussion

### Main Findings and Interpretation

The main finding of the present study is that the primary analysis did not identify associations between CT-derived radiologic lesion characteristics and pain, while in some secondary outcomes significant relationships were found. These findings indicate that the relationship between lesion characteristics is highly complex. For the primary outcome, the FAOS Pain subscale, worse pain scores were associated with higher age and smoking.

To our knowledge, this is the first study to investigate the relationship between baseline patient and lesion characteristics and pain severity, measured primarily by the FAOS Pain subscale and secondarily by the NRS. An editorial by van Dijk et al.^7^ hypothesized that pain in OLTs results from repeated elevations in synovial fluid pressure stimulating the innervated subchondral bone plate, with cartilage itself playing no direct role. Based on this theory, lesion depth and cystic morphology were expected to be important contributors. However, in our study, lesion depth was only associated with pain at rest—an unexpected finding, given that synovial fluid pressure is presumed lower at rest than during activity.

Smoking emerged as a modifiable risk factor: smokers reporting higher pain levels.^29^ Although the observed difference of 8.7 points does not exceed the minimal important change,^30^ the biological plausibility is strong. Smoking induces low-grade inflammation,^31,32^ is associated with impaired vascularization and delayed healing,^33,34^ and often clusters with psychosocial and behavioral risk factors.^35^ Together, these may contribute to heightened pain perception.

In one sub-analysis, a modest association was found between lesion depth and NRS pain during rest. As this association was only found in this model, with a low explained variance, caution is needed when interpretating this. Moreover, pain during rest is not typical for OLTs, which typically cause deep ankle pain during activity/weightbearing. In the sub-analysis for NRS during walking, fragmentary morphology showed to be associated with a lower score (ie, less pain). No clear reason could be identified for this intriguing finding. However, as these findings are not consistent across the different scores, they should be considered as hypothesis-generating and needs further research in the future.

The lack of a consistent association between radiologic lesion characteristics and pain supports the notion that pain in OLTs is driven by more than structural damage alone. Psychosocial and systemic influences likely play a role.^9^ This aligns with the frequent occurrence of asymptomatic OLTs and the underestimation of their prevalence.^5^ These findings highlight the potential value of a multidisciplinary approach to pain management. Future research should further explore systemic contributors—including metabolic health, inflammatory markers, and psychosocial domains—and investigate whether advanced imaging techniques, such as functional magnetic resonance imaging (fMRI) or 3D volumetric segmentation, can detect more subtle structural correlates of pain.

### Strengths and Limitations

This study has limitations. The explained variance of the multivariate models was relatively low, indicating that other unmeasured factors influence pain perception in OLT patients. Consequently, the models are not suitable for clinical prediction. Another limitation is that this study was CT-based and did not include MRI variables. Therefore, MRI-derived variables such as bone marrow edema and soft tissue changes were not assessed in the present study. However, CT is widely used and provides a more adequate view on the structural changes in the subchondral bone. Future studies using MRI could be conducted to analyze the relationship between MRI findings (eg, bone marrow edema, effusion, synovitis) and pain in OLTs. In this respect, it should also be mentioned that multiple potential confounders (eg, symptom duration, analgesic use, and psychological factors) could not be assessed as there is a limit of variables that can be included with sufficient power. Furthermore, treatment outcomes were not assessed in relation to baseline characteristics. Future longitudinal studies should investigate whether modifiable factors affect both pain and treatment response. Lastly, the present study used a cohort from a tertiary referral center. Therefore, the results might not be generalizable to other centers and clinical application is not feasible yet.

Strengths include the large sample size (318 patients), the robustness of a complete-case sensitivity analysis, and the independent evaluation of CT scans by 2 researchers, ensuring high-quality radiologic assessment.

### Clinical Relevance

These findings emphasize that radiographic lesion characteristics do not reflect pain severity in OLT patients. Clinical assessment should therefore avoid tunnel vision and adopt a patient-centered, multidisciplinary approach.

## Conclusion

No association was found between CT-based radiologic lesion characteristics and patient-reported pain as measured by the FAOS Pain subscale. Therefore, structural damage alone does not seem to fully explain the patient’s pain, reinforcing the orthopaedic adage: *treat the patient, not the scan*. Importantly, the present study has limitations and therefore further research is needed.

## Supplemental Material

sj-pdf-1-fai-10.1177_10711007251413568 – Supplemental material for Understanding Pain in Osteochondral Lesions of the Talus: A Cross-Sectional, CT‑Based Analysis Showing Limited and Inconsistent Associations With Pain Supplemental material, sj-pdf-1-fai-10.1177_10711007251413568 for Understanding Pain in Osteochondral Lesions of the Talus: A Cross-Sectional, CT‑Based Analysis Showing Limited and Inconsistent Associations With Pain by Julian J. Hollander, Inger N. Sierevelt, Jason A. H. Steman, Juliëtte H. M. Pijnacker, Kaj S. Emanuel, Gino M. M. J. Kerkhoffs and Sjoerd A. S. Stufkens in Foot & Ankle International
